# Letter to the Editor on “Analgesic Effects of Ketamine, Magnesium Sulfate, and Sodium-Thiopental on Propofol Injection Pain: A Single-Blind Randomized Clinical Trial”

**Published:** 2019-02

**Authors:** Reza Aminnejad

**Affiliations:** Department of Anesthesiology & Critical Care, Qom University of Medical Sciences, Qom, Iran, Department of Pain Medicine, Shahid Beheshti University of Medical Sciences, Tehran, Iran

Dear Editor,

I was searching for new modalities to prevent propofol injection pain (PIP) and I encountered and read with interest an article written by Akbari et al. in your journal (1). First of all, it seems that units used for injected ketamine and sodium thiopental is incorrect and they should be mg/Kg not ml/Kg. In our daily clinical practice as an anesthesiologist, we use 5% solution of sodium thiopental (50 mg/ml) and every anesthesia team member has experienced that injecting this concentration is per se painful in comparison to 2.5% solution. As a routine practice, anesthesiologists used to administer propofol slowly via a large bore intravenous access or following injection of a minimal dose of lidocaine (1 to 2 ml of 2% solution) (2). The important issue regarding PIP is the importance of it. PIP is not an important problem and it is not a common source of bitter memories of surgery patients (3). From this point of view, adding unnecessary medications to the anesthesia regimen of an elective patient may expose him/her to unwanted risks. Propofol injection is painful and there is no doubt about it. But in clinical practice we should consider about costs and benefits of adding extra-drugs to usual regimens. If you suggest using combination of drugs such as ketamine or sodium thiopental, occurring unwanted events such as postoperative cognitive disorders or PONV are inevitable (4, 5).
